# Blood count, endocrine, immunologic, renal, and hepatic markers in a case-control animal study of induced periodontitis in female rodents

**DOI:** 10.3389/fphys.2024.1327399

**Published:** 2024-02-20

**Authors:** João Estarreja, Ana Clara Pimenta, João Botelho, Arminda Maria Vilares, José João Mendes, João Rocha, Rui Pinto, Vanessa Mateus, Vanessa Machado

**Affiliations:** ^1^ H&TRC—Health and Technology Research Center, ESTeSL—Escola Superior de Tecnologia da Saúde de Lisboa, Instituto Politécnico de Lisboa, Lisbon, Portugal; ^2^ Clinical Research Unit (CRU), Egas Moniz Center for Interdisciplinary Research, Egas Moniz School of Health & Science, Almada, Portugal; ^3^ Laboratório de Fisiologia e Bioquímica do Exercício, Universidade de Lisboa Faculdade de Motricidade Humana, Lisboa, Portugal; ^4^ iMed.ULisboa, Faculdade de Farmácia, Universidade de Lisboa, Lisbon, Portugal; ^5^ Joaquim Chaves Saúde, Joaquim Chaves Laboratório de Análises Clínicas, Miraflores, Portugal

**Keywords:** periodontitis, inflammation, disease animal models, rodents, non-clinical study *in vivo*

## Abstract

**Introduction:** Periodontitis is a non-communicable chronic inflammatory disease with a systemic burden. Animal models of induced periodontitis help elucidate the mechanisms by which periodontal inflammation drives systemic effects. Studying this systemic involvement over longer follow-up periods may provide a strong foundation for future research on the association between diseases and periodontitis, particularly in female rats. Therefore, we aimed to compare blood, endocrine, immunologic, renal, and hepatic markers in a rat model of induced periodontitis in females with their control counterparts.

**Methods:** Experimental periodontitis was induced in 20 female Wistar rats by the application and maintenance of silk ligatures on the upper molars. The rats were then assessed for macroscopical analysis, complete blood count, and biochemical, endocrine, and immunologic markers at 21, 28, 42, and 56 days.

**Results:** Chronic periodontal inflammation was observed after 42 days of exposure to the ligatures. Additionally, it was also possible to notice significant systemic manifestations, such as the reduction of triiodothyronine and thyroxine levels, along with an increase in the expression of alkaline phosphatase, gamma-glutamyl transpeptidase, and lactate dehydrogenase.

**Discussion:** The study’s findings imply that certain changes can be underscored to highlight a reduced risk of conception. Notably, previous investigations have indicated that subfertile women exhibit lower levels of thyroid hormones and elevated lactate dehydrogenase expression. Despite the absence of preclinical data delineating a possible association between periodontitis and female infertility, the results of this study may prove to be a crucial contribution to both the scientific and medical fields.

## 1 Introduction

Periodontitis is a chronic non-communicable inflammatory disease characterized by the progressive destruction of tooth-supporting tissues due to a complex interplay between the oral microbiome and exacerbated reaction of the host immune system against the periodontium (Hajishengallis, 2023). Nowadays, periodontitis is a global public health problem affecting, approximately, 60% of the adult dentate population and has increased over the last 30 years, resulting in significant economic repercussions (Chen et al., 2021; Botelho et al., 2022; Trindade et al., 2023).

Deterioration of the periodontium allows invasion of periodontal pathogens through the bloodstream and triggers a systemic inflammatory response that can affect any tissue in the body (Lang and Bartold, 2018). Recently, periodontal disease has been strongly associated with an increased risk of several systemic diseases, including adverse pregnancy outcomes, polycystic ovarian syndrome, and altered levels of mean corpuscular hemoglobin and serum C-reactive protein (CRP) (Machado et al., 2023). Furthermore, periodontal health in women during reproductive years is an important component of health and reduces the risk of conceptions difficulties (Hart et al., 2012; Machado et al., 2020a; Telatar et al., 2021). There are a few reasons to support this rationale: i) periodontitis is highly prevalent in this population (Chen et al., 2021; Trindade et al., 2023); ii) 10%–20% of women with infertility have idiopathic causes (Sunderam et al., 2018); iii) periodontitis is strongly related to common pathways associated with hormonal abnormalities, such as inflammation, infection, metabolic disorders, decidual dysfunction and vascular disorders, provoking an increase in inflammatory mediators in the female reproductive system and placenta, which can elevate the risk of non-conception and adverse pregnancy outcomes (Madianos et al., 2013; Vannuccini et al., 2016; Martelli et al., 2017; Mainas et al., 2022), and iv) periodontitis has been implicated in a bidirectional association with polycystic ovarian syndrome (Machado et al., 2020b). Despite these findings, the causal relationship and underlying biological mechanisms involved in periodontitis and female infertility are not fully understood.

Animal models have played a pivotal role in advancing mechanistic understanding, although no single model can fully replicate all aspects of periodontitis in humans (Hajishengallis, 2023). Notwithstanding, the major benefits are the ability to help advance scientific discovery in a variety of medical fields, conduct longitudinal studies from the initial onset to the progression of lesions in multiple tissues, and control for confounding variables.

Considering the lack of preclinical evidence on the link between periodontitis and female infertility, this study aimed to map the inflammatory and endocrine implications of periodontitis in the blood and reproductive tissues of female rat model of periodontitis.

## 2 Materials and methods

### 2.1 Animals

Twenty-eight female Wistar rats (8–10 weeks, 200 g ± 30 g) were used throughout the experiment. Animals were allowed 1 week to acclimate and were housed in standard polypropylene cages, with access to food and water *ad libitum*. Temperature (18ºC–23°C), humidity (40%–60%), and light (12-h day/night cycle) were controlled during the experiment. Animal care was in accordance with the internationally accepted principles for the use and care of laboratory animals, found in Directive 2010/63/EU (Union, 2010). The experimental protocol was approved by the Ethics Committee for Animal Experimentation of the Faculty of Pharmacy of the University of Lisbon (process number 6/2021). The experiment was conducted in a pharmacology laboratory at the Faculty of Pharmacy, University of Lisbon (Portugal).

### 2.2 Induction of periodontitis

The rats were randomly divided into four experimental groups of five rats each, and two rats were used in the respective control groups. The experimental groups had their both maxillary second molars tooth ligated and then were sacrificed at four different time points, after ligation (days 21, 28, 42 and 56). Before each experimental procedure, the rats were anesthetized with a solution consisting of ketamine 100 mg/kg (Ketamidor^®^ 10%, Merial^®^) and xylazine 10 mg/kg (Sedaxylan^®^ 2%, Bayer^®^), by intraperitoneal injection. To induce bone loss, a 5–0 silk ligature (Strategic Material, Inc^®^) was placed submarginally, and maintained, around the maxillary second molars. We applied silk ligatures to promote the local accumulation of oral bacteria, as the microfilaments present increases the surface area for attachment. This led to an increase bacteria-mediated inflammation and contributed to bone loss (Abe and Hajishengallis, 2013; Tomina et al., 2022; Khuda et al., 2024). Throughout the experimental period, the rats were anesthetized every 2 weeks to ensure that the ligatures were present, and in case of loss, they were reapplied. Rats in the respective control groups were not subjected to any experimental procedures.

### 2.3 Maxillary dissection and macroanalyses

At four different time points after ligation, the rats were anesthetized to collect the samples of interest. The animals were initially kept alive for cardiac puncture to collect the blood samples to determine the concentration of hematological, biochemical, endocrine, and immunological markers. The rats were euthanized via cervical dislocation. The maxilla of each animal was dissected, and upper gingiva was carefully removed. Following gingival dissection, bone samples were immersed in 3% hydrogen peroxide, for 24 h, to facilitate the removal of all remaining soft tissues. Subsequently, the samples were stored in 70% ethanol. On the day of macroscopic evaluation, the collected gingival samples were dried and immersed in methylene blue (0.7 g/L), for 5 min, and then the samples were washed with water to remove the additional methylene blue stain (Sigma-Aldrich^®^) (de Molon et al., 2018). The buccal and lingual surfaces of the stained specimens were digitally photographed using a stereomicroscope (Leica Microsystems^®^, Germany, ×20 magnification). A calibrated and blinded examiner (V.Machado) used ImageJ (Image Tool 3.0 software program, Department of Dental Diagnostics Science, University of Texas Health Science Center, San Antonio, TX, United States), for periodontal measurements. The cemento-enamel junction (CEJ) to alveolar bone crest (ABC) distance was measured at six locations per tooth (distal-buccal, buccal, mesio-buccal, distal-lingual, lingual, and mesio-lingual).

### 2.4 Quantification of systemic biomarkers

After performing cardiac puncture in each rat, the total blood sample was divided into separate tubes, of approximately 1,800–2,000 µL each. To obtain a complete blood count, a portion of the blood sample was placed in a BD Vacutainer^®^ tube (VWR^®^) with EDTA K2 additive to prevent clotting. The samples were then analyzed using an automated hematology analyzed (Sysmex XN-10TM, Sysmex^®^), to quantity the expression of the different cell types. On the other hand, to analyze the expression of biochemical, endocrine, and immunological markers, the other part of the blood sample was placed in a BD Vacutainer^®^ SSTTM II advance tube containing a polymer gel separator. The samples were separated by centrifugation (3000xg for 5 min) and the serum was analyzed on a modular analyzer (Cobas^®^ 8,000, Roche^®^) using appropriate C702, E801, and ISE modules.

### 2.5 Statistical analysis

Continuous variables were expressed as the mean (standard deviation) of N observations, where N represents the number of animals analyzed. Data analysis was performed using GraphPad 5.0 software (GraphPad, San Diego, CA, United States). The results were analyzed either by *t*-test or one-way ANOVA, depending on the outcomes, to determine statistical significance between the experimental and control groups, followed by Tukey’s *post hoc* test for multiple comparisons. A *p*-value of 0.05 was considered to be significant.

## 3 Results

### 3.1 Clinical signs

**FIGURE 1:**
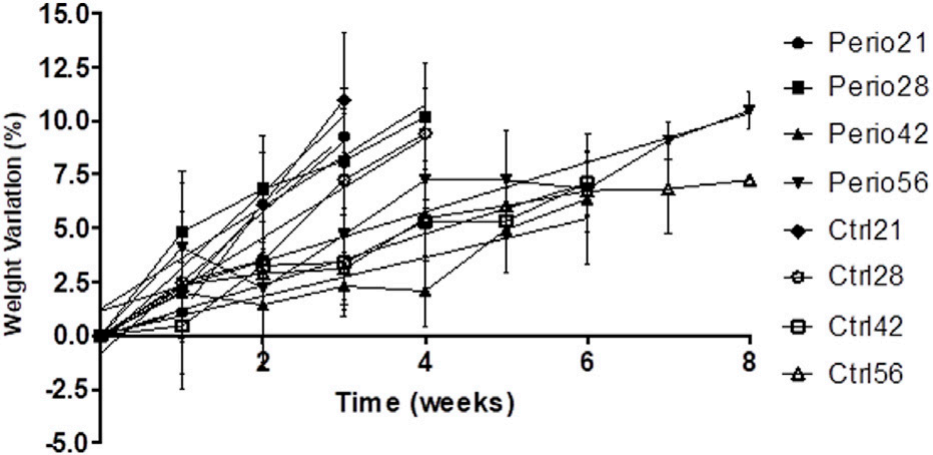
Body weight variation in animals.

An important indicator of the safety of experimental rats is the change in body weight, general appearance, and behavior after induction of periodontitis. No negative changes were observed among the experimental groups. All the rats showed an increase in body weight during the experimental procedures (Figure 1), and the final values were similar between the experimental and control groups. Indeed, it was not possible to identify any significant differences between the groups (*p* > 0.05). Curiously, it was observed more similarities between the groups of 21 and 28 days (Perio21 = +9.27% ± 4.34%; Ctrl21 = +10.98% ± 4.84%; Perio28 = +10.19% ± 3.88%; Ctrl28 = +9.42% ± 3.78%), when compared to the others with a longer time spectrum (Perio42 = +6.36% ± 2.17%; Ctrl42 = +7.09% ± 2.62%; Perio56 = +10.47% ± 3.33%; Ctrl56 = +7.24% ± 2.51%).

### 3.2 Macroscopical periodontal analysis

**FIGURE 2:**
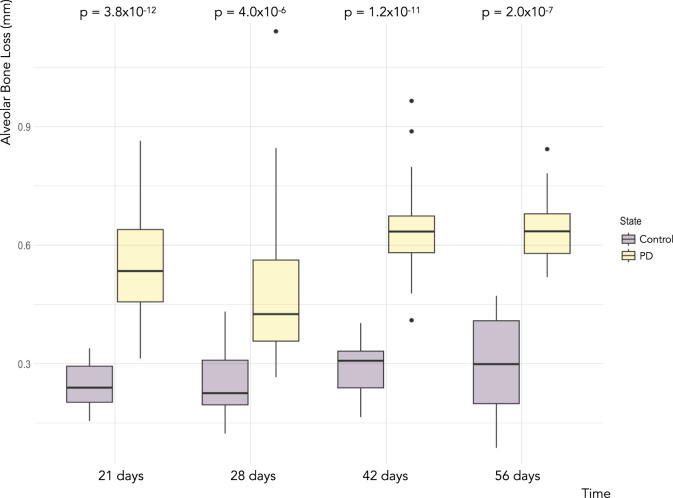
Boxplot of alveolar bone loss for the different measurements of the linear distance between the CEJ and ABC, on the buccal and lingual surfaces, in the maxillary second molar in the periodontitis induction model and respective control groups. Legend: Boxes indicate the 25th and 75th percentiles, error bars indicate the 5th and 95th percentiles, circles show outliers, and solid horizontal lines show the median.

Statistically significant differences were identified in the overall values obtained between the groups related to the distance between the CEJ and ABC. As Figure 2 shows, it is possible to notice significantly higher distance values in the periodontitis groups than in the respective controls, where the timeline is equal. Quantitatively, it was noticed a significant increase in the distance values in the Perio21 group (0.56 mm ± 0.12 mm), when compared to the Ctrl21 (0.26 mm ± 0.02 mm) and Ctrl28 groups (0.25 mm ± 0.03 mm). In addition, both Perio42 (0.64 mm ± 0.07 mm) and Perio56 (0.65 mm ± 0.04 mm) groups showed significantly higher distance values, in comparison to the Ctrl21 (0.25 mm ± 0.02 mm), Ctrl28 (0.25 mm ± 0.03 mm), Ctrl42 (0.29 mm ± 0.02 mm), and Ctrl56 (0.29 mm ± 0.07 mm) groups. The shortest distance between periodontitis groups was observed in the Perio28 group (0.49 mm ± 0.16 mm).

**FIGURE 3:**
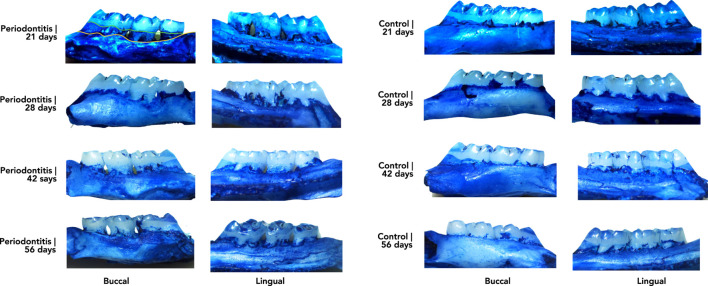
Illustrative representation of the gingival tissue in the periodontitis and control groups related to the development of the ligature-induced rat model of periodontitis. Legend: Left photos represent the gingival tissue from the rats subjected to the induction of periodontitis, organized vertically from the Perio21 group (top) until the Perio56 group (bottom). Photographs on the right represent the gingival tissue of control rats organized vertically from the Ctrl21 group (top) to the Ctrl56 group (bottom).

Considering the illustrative representation of the gingiva in the animals present in the periodontitis and control groups (Figure 3), it is possible to identify a substantial destruction, related to the inflammatory response, in the Perio21 group, which decays in the next 7 days, as represented in the Perio28 group, although without statistical significance. Furthermore, regarding the Perio42 and Perio56 groups, there was a similar grade of destruction between them, which once more ascertained the quantitative data, previously referred.

### 3.3 Complete blood count

The complete blood count values obtained demonstrated no significant differences between the periodontitis groups, considering the different timelines adopted (Table 1). In addition, there was a lack of statistical significance in the differences observed between the periodontitis and control groups, where the timeline is equal. However, there was a significant increase in neutrophils in the Perio56 group compared to the Ctrl28 group (25.23% ± 5.93% vs. 6.95% ± 2.19%, *p* < 0.05). The highest value concerning the concentration of this cell type was observed in Perio56 group. Furthermore, considering the values reached for this parameter, it was also possible to observe that the periodontitis groups demonstrated an increased concentration when compared to the controls, which was expected at the beginning. However, with respect to the concentration of basophils, it was only possible to notice an increased concentration in Perio21 and Perio42 groups, in comparison to the null values obtained in the other experimental and control groups, however this was not statistically significant.

**TABLE 1:** Complete blood count values obtained from the animals.

Days	Periodontitis				Control			
	21	28	42	56	21	28	42	56
Red Blood Cells (10^9^/L)	7.36 (0.33)	7.71 (0.19)	7.77 (0.35)	7.60 (0.44)	6.98 (0.76)	7.66 (0.45)	7.26 (0.44)	7.66 (0.91)
White Blood Cells (10^12^/L)	2.18 (0.52)	1.67 (0.69)	1.65 (0.52)	1.73 (0.75)	2.19 (0.74)	2.78 (2.41)	1.40 (0.68)	1.22 (0.20)
Hemoglobin (g/dL)	13.40 (0.48)	14.08 (0.54)	14.28 (0.70)	14.03 (0.67)	13.05 (2.05)	14.50 (0.85)	13.55 (1.06)	14.15 (1.20)
Hematocrit (%)	48.24 (2.96)	47.84 (1.45)	49.03 (2.29)	52.93 (2.40)	44.40 (7.50)	47.85 (2.90)	45.85 (3.23)	53.85 (3.61)
Platelets (10^9^/L)	627.60 (131.20)	542.40 (216.60)	522.00 (78.21)	512.00 (313.30)	473.50 (294.90)	519.00 (309.70)	467.50 (280.70)	782.00 (18.38)
Neutrophils (%)	15.76 (4.68)	13.10 (8.62)	14.18 (4.01)	25.23 (5.93)*	8.05 (0.50)	6.95 (2.19)*	8.35 (2.05)	22.25 (3.89)
Lymphocytes (%)	82.62 (4.40)	84.94 (9.88)	83.80 (4.38)	72.87 (5.54)	90.10 (0.42)	91.50 (2.55)	89.75 (2.48)	76.55 (4.31)
Macrophages (%)	1.28 (0.33)	1.22 (0.64)	1.35 (0.70)	1.10 (0.50)	1.35 (0.21)	0.90 (0.00)	1.65 (0.78)	1.20 (0.42)
Eosinophils (%)	0.20 (0.31)	0.64 (0.91)	1.35 (0.70)	0.70 (0.36)	0.50 (0.14)	0.65 (0.35)	0.25 (0.35)	1.20 (0.42)
Basophils (%)	0.14 (0.31)	0.00 (0.00)	0.28 (0.55)	0.00 (0.00)	0.00 (0.00)	0.00 (0.00)	0.00 (0.00)	0.00 (0.00)

**Legend:** *p < 0.05, compared between respective groups. The results are presented as the mean (standard deviation) [n (Perio21 and Perio28 groups): 5 rats; n (Perio42 group): 4 rats; n (Perio56 group): 3 rats; n (Ctrl21, Ctrl28, Ctrl42, and Ctrl56 groups): 2 rats]. Statistical analysis was performed using one-way analysis of one-way ANOVA, and Tukey’s *post hoc* test.

### 3.4 Endocrine biomarkers

Regarding the concentrations of cortisol and sex-related hormones, namely, estradiol and progesterone, no statistically significant differences were observed between the experimental and control groups (Table 2). In Table 2, the values of cortisol reached in all groups are quite similar between them. The highest value was obtained in the Ctrl42 group. Furthermore, there was a reduction in estradiol levels in all periodontitis groups compared with the respective controls. The lowest levels of this hormone were observed in the Perio42 group. Concerning the concentration of progesterone, the results are paradoxical, since the Perio21 and Perio28 groups showed a pronounced reduction in this hormone compared to the other two periodontitis groups. The values reached in the Perio42 and Perio56 groups were substantially higher than those in the respective controls, considering the same timelines. Finally, the evaluation of the thyroid-related hormones, triiodothyronine (T3) and thyroxine (T4), demonstrated significant differences between the groups. Indeed, considering the concentration of T3, it was possible to observe a significant reduction in its levels in the Perio42 and Perio56 groups compared to the Perio28 group (*p* < 0.05). Similarly, there was a pronounced reduction in the concentration of T4 in the Perio42 and Perio56 groups compared to other two periodontitis groups. However, it was only possible to confirm a significant reduction in this hormone in the Perio42 group compared to the Perio21, Perio28, and Ctrl21 groups (*p* < 0.05).

**TABLE 2:** Expression of endocrine biomarkers in animals.

Days	Periodontitis				Control			
	21	28	42	56	21	28	42	56
Cortisol (ng/mL)	2.3 (0.70)	2.26 (0.55)	2.85 (0.39)	2.99 (0.37)	2.20 (0.28)	2.90 (0.99)	3.40 (0.42)	2.90 (0.14)
Estradiol (ng/mL)	14.58 (13.29)	14.94 (12.71)	7.78 (2.32)	19.90 (8.68)	24.40 (24.89)	25.30 (22.34)	21.00 (11.31)	29.50 (2.12)
Progesterone (ng/mL)	9.84 (3.01)	9.02 (4.32)	16.13 (3.23)	16.30 (1.35)	14.15 (9.69)	9.70 (3.25)	14.70 (1.70)	10.85 (1.91)
T3 (ng/dL)	123.20 (15.35)	135.00 (13.58)	99.15 (8.37)*	98.00 (6.93)*	124.00 (7.07)	-	106.00 (24.04)	101.50 (10.61)
T4 (µg/dL)	4.10 (0.44)#	4.16 (0.70)#	2.73 (0.25)	3.22 (0.40)	4.30 (0.57)#	-	3.15 (0.78)	2.95 (0.35)

**Legend:** T3, triiodothyronine; T4, thyroxine. *p < 0.05, compared to the Perio28 group. #*p* < 0.05, compared to the Perio42 group. Results are presented as the mean (standard deviation) [n (Perio21 and Perio28 groups): 5 rats; n (Perio42 group): 4 rats; n (Perio56 group): 3 rats; n (Ctrl21, Ctrl28, Ctrl42, and Ctrl56 groups): 2 rats]. Statistical analysis was performed using one-way ANOVA, and Tukey’s *post hoc* tests.

### 3.5 Immunological markers

The evaluation of systemic inflammatory and immunological repercussions during the development of the ligature-induced rat model of periodontitis demonstrated that there were no significant differences between the experimental and control groups, in most cases (Table 3). Indeed, the unique difference with statistical significance was noticed in the concentrations of IgA between the control groups, namely, Ctrl28 and Ctrl56 (*p* < 0.05). Considering the concentrations of CRP, the highest value was obtained in the Perio42 group and the lowest in the Ctrl28 group. Regarding the expression of immunoglobulins (Igs), similar concentrations of IgA were observed among all groups, with the highest level identified in the Ctrl28 group. Additionally, we observed an increase in IgG values in the Perio42 group, which was the highest value reached, compared with the other periodontitis and control groups. Considering this parameter, it can be concluded that the experimental groups had higher concentrations than the controls, in most cases. Furthermore, the concentration of IgM was generally higher in the periodontitis groups, with the maximum value found in the Perio21 group.

**TABLE 3:** Immunological biomarker expression in animals.

Days	Periodontitis				Control			
	21	28	42	56	21	28	42	56
CRP (μg/dL)	3.40 (4.67)	5.00 (5.39)	11.50 (6.86)	9.67 (12.66)	6.00 (5.66)	0.00 (0.00)	9.50 (12.02)	4.50 (4.95)
IgA (mg/dL)	1.46 (0.31)	1.14 (0.26)	1.30 (0.41)	1.00 (0.00)	0.90 (0.00)	1.80 (0.42)*	1.00 (0.00)	0.55 (0.64)*
IgG (mg/dL)	93.32 (20.70)	73.38 (8.45)	145.10 (19.83)	108.70 (51.39)	98.50 (13.44)	59.50 (0.71)	65.20 (19.37)	93.05 (4.17)
IgM (mg/dL)	6.70 (3.29)	6.44 (2.12)	6.48 (1.48)	4.07 (0.90)	5.00 (1.13)	5.85 (0.35)	6.20 (0.71)	3.70 (1.98)

**Legend:** CRP, C-reactive protein; IgA, Immunoglobulin A; IgG, Immunoglobulin G; IgM, Immunoglobulin M. *p < 0.05, compared between the respective groups. Results are presented as the mean (standard deviation)[n (Perio21 and Perio28 groups):5 rats; n (Perio42 group):4 rats; n (Perio56 group):3 rats; n (Ctrl21, Ctrl28, Ctrl42, and Ctrl56 groups):2 rats]. Statistical analysis through the application of one-way ANOVA, and Tukey’s *post hoc* tests.

### 3.6 Renal and hepatic functions

Renal and hepatic markers revealed no statistically significant differences between the experimental and control groups (Table 4). Considering the evaluation of renal function, it was possible to observe that the concentrations of urea and creatinine were quite similar between all groups. The highest concentrations of these biomarkers were found in the Perio56 group. In contrast, considering liver function, pronounced variations in both aspartate transaminase (AST) and alanine aminotransferase (ALT) levels were observed between the experimental and control groups. Indeed, there was a pronounced increase in AST levels in the Perio28 group, when compared with the other periodontitis groups. Overall, the concentrations of AST were higher in the experimental groups than in the control groups. Regarding the expression of ALT, the highest values obtained were also found in the Perio28 and Ctrl28 groups (Table 4).

**TABLE 4:** Renal and hepatic biomarker expression in animals.

Days	Periodontitis				Control			
	21	28	42	56	21	28	42	56
Urea (mg/dL)	42.40 (2.30)	42.60 (4.34)	43.00 (3.46)	46.07 (3.00)	46.00 (1.41)	45.50 (0.71)	45.50 (2.12)	33.15 (2.62)
Creatinine (mg/dL)	0.47 (0.05)	0.48 (0.02)	0.50 (0.04)	0.54 (0.02)	0.46 (0.01)	0.50 (0.02)	0.45 (0.02)	0.46 (0.06)
AST (U/L)	150.20 (42.70)	234.60 (73.91)	93.75 (10.14)	129.00 (16.52)	163.50 (13.44)	210.00 (91.92)	81.00 (4.24)	77.00 (25.46)
ALT (U/L)	35.80 (6.26)	49.60) (21.62)	45.15 (4.28)	47.53 (5.76)	38.50 (2.12)	54.00 (16.97)	42.50 (4.95)	26.50 (3.54)

**Legend:** ALT, alanine aminotransferase; AST, aspartate transaminase. Results are presented as the mean (standard deviation)[n (Perio21 and Perio28 groups):5 rats; n (Perio42 group):4 rats; n (Perio56 group):3 rats; n (Ctrl21, Ctrl28, Ctrl42, AND, Ctrl56 groups):2 rats]. Statistical analysis through the application of one-way ANOVA, and Tukey’s *post hoc* tests.

### 3.7 Other biochemical markers

Looking at other biomarkers, we observed additional significant differences (Table 5). The expression of alkaline phosphatase (ALP), at 21 days of periodontitis was significantly higher values than that at 28 (*p* < 0.01), 42 (*p* < 0.001), and 56 days (*p* < 0.001). In addition, compared with the controls, the values reached in the Perio21 group were significantly higher than those in the Ctrl42 (*p* < 0.01) and Ctrl56 (*p* < 0.01) groups.

**TABLE 5:** Biochemical marker expression in animals.

Days	Periodontitis				Control			
	21	28	42	56	21	28	42	56
Albumin (g/dL)	4.32 (0.22)	4.33 (0.29)	4.78 (0.30)	4.93 (0.15)	4.45 (0.07)	4.55 (0.35)	4.80 (0.14)	4.80 (0.56)
ALP (U/L)	108.20 (25.33)	60.60 (11.55)**	46.28 (7.73)***	42.57 (10.77)***	68.50 (13.44)	65.50 (26.16)	31.50 (2.12)**	33.00 (0.00)**
Amilase (U/L)	1,697.00 (258.00)	1763.00 (238.90)	1,691.00 (65.77)	1,661.00 (243.50)	1,587.00 (144.20)	1,544.00 (103.90)	1889.00 (18.38)	1,636.00 (157.70)
Amilase P (U/L)	1,439.00 (211.80)	1,543.00 (167.70)	1,462.00 (53.80)	1,442.00 (201.30)	1,345.00 (111.00)	1,359.00 (0.00)	1,689.00 (29.70)	1,446.00 (164.80)
Creatine Kinase (U/L)	1,337.00 (576.10)	1,148.00 (392.70)	410.50 (122.40)*	504.30 (153.60)	1,112.00 (181.70)	1,330.00 (112.40)	333.50 (17.68)	398.50 (26.16)
GGT (U/L)	-	-	0.63 (0.86)	0.10 (0.00)^a^	-	-	0.10 (0.00)^a^	0.10 (0.00)^a^
Iron (ug/dL)	419.00 (45.74)	424.30 (78.39)	464.00 (34.56)	495.30 (34.59)	478.50 (54.45)	423.00 (14.14)	502.50 (19.09)	460.00 (12.73)
LDH (U/L)	964.80 (293.20)	987.00 (82.25)	678.50 (188.00)	610.30 (51.43)	1,073.00 (74.95)	860.50 (218.50)	396.00 (8.49)*#$	382.00 (26.87)*#$
Lipase (U/L)	6.32 (0.15)	5.97 (0.38)	5.98 (0.33)	4.47 (2.25)	5.85 (0.07)	5.60 (0.00)	6.35 (0.21)	4.95 (0.07)
Total proteins (g/dL)	5.90 (0.55)	6.06 (0.29)	6.45 (0.21)	6.57 (0.12)	6.15 (0.07)	6.20 (0.28)	6.35 (0.21)	6.35 (0.50)

**Legend:** ALP, alkaline phosphatase; GGT, gamma-glutamyl transpeptidase; LDH, lactate dehydrogenase. *p < 0.05; **p < 0.01, ***p < 0.001, in comparison to the Perio21 group. #*p* < 0.05, compared to the Perio28 group. $*p* < 0.05, compared to the Ctrl21 group.

^a^ p < 0.05, in comparison to the Perio42 group. Results are presented as the mean (standard deviation) [n (Perio21 and Perio28 groups) 5 rats; n (Perio42 group) 4 rats; n (Perio56 group) 3 rats; n (Ctrl21, Ctrl28, Ctrl42, and Ctrl56 groups) 2 rats]. Statistical analysis through the application of one-way ANOVA, and Tukey’s *post hoc* tests.

Additionally, the expression of creatine kinase (CK) was significantly increased at 21 days of periodontitis compared to 42 days of periodontitis (*p* < 0.05).

The expression of gamma-glutamyl transpeptidase (GGT) was only available for 42 and 56 days of each periodontitis and control groups, and was significantly increased at 42 days of periodontitis, compared to the rest of the groups.

The concentration of lactate dehydrogenase (LDH)was highest at 21 days in the control group. After 21 and 28 days of periodontitis, the levels of this biomarker were higher than those in the corresponding control groups (*p* < 0.05).

Regarding the remaining biomarkers, there were no significant differences among the groups (*p* > 0.05).

## 4 Discussion

Throughout the development of our rat model of periodontitis, we evaluated the morphometric and serum manifestations at different stages of periodontitis, over a period spectrum of 56 days. Persistent periodontal inflammation was noted, along with decreased levels of estradiol, T3, and T4, and increased levels of ALP, CK, GGT, and LDH.

The comprehensive characterization of existing animal models of periodontitis allows to mimic its clinical reality in humans (Abe and Hajishengallis, 2013; de Molon et al., 2018; Hajishengallis, 2023). In this effort, we used different time points to describe the model, specifically during its chronicity. Macroscopic analysis revealed bone resorption in the furcation and interproximal regions in all ligated animals, mimicking what seems to occur in humans. During the development of this rat model, the acute phase of periodontitis was clearly observed 21 days after ligature placement, resulting in a pronounced destruction of the periodontium. Similarly, to previous studies, periodontal tissues tend to migrate to a more apical position to regain biological space and reduce the severity of periodontitis over time (de Molon et al., 2014; de Molon et al., 2016).

To evaluate the severity of the disease over time, we also evaluated alveolar bone loss at 42 and 56 days. At 42 days of ligature exposure, a more severe alveolar bone destruction was observed, following a stabilization of bone resorption, mimicking a chronic phase of periodontitis in humans. This is of particular significance, as it has been demonstrated through clinical evidence that the most severe stage of periodontitis is linked to the greatest risk of female infertility (Machado et al., 2020c). The time of retention of the ligatures in the teeth is directly proportional to the alveolar bone destruction observed (Zhang et al., 2023). This may be explained by the fact that bacteria continue to accumulate in ligatures over time, causing a chronic inflammatory response that leads to a more severe stage of periodontitis.

Serum inflammatory and immunological mediators were important to understand the level of response against the ligature placement (Gomes-Filho et al., 2011; Zhang and Deng, 2019; Machado et al., 2021). Our results showed a marked difference in the concentration of neutrophils. Increased concentrations of these immune cells were observed in the periodontitis groups, which is consistent with previous studies (Cintra et al., 2014; Tomina et al., 2022). The increase in neutrophil expression may be associated with the development of an exacerbated inflammatory response in the periodontium, being even noticed systematically.

Regarding thyroid-related hormones, it has been postulated that serum T3 and T4 levels are normally lower in the case of disease, and a gradual decrease is observed with increasing severity (Wenzek et al., 2022). In fact, a significant reduction in T3 and T4 levels was observed in the Perio42 and Perio56 groups compared to the other two timelines adopted, which was expected given the above statement. Therefore, it is possible to identify a potential negative influence of periodontitis on normal functioning of the thyroid. In addition, the reduction of these two hormones may influence female fertility since it has been described that infertile women usually present lower serum levels of T3 and T4 (Bassey et al., 2015).

An overall biochemical marker evaluation is essential to investigate the potential relationship between two or more different conditions, when the underlying mechanisms remain unclear. Given the exploratory nature of this study, other biochemical markers were also evaluated. The measurement of ALP, an enzyme involved in periodontal destruction, showed significant differences between the experimental and control groups (Sanikop et al., 2012). Twenty-one days after ligature placement, a significant increase in the concentration of ALP was noted compared to the other periodontitis and control groups. These results are consistent with the literature, as in the early stages of periodontitis, the concentration of this biomarker is normally elevated due to the extensive tissue destruction observed (Malhotra et al., 2010; Jeyasree et al., 2018; Koppolu et al., 2021).

Concerning the concentration of CK, an enzyme involved in energy metabolism and commonly used to screen for muscle damage, it was expected to observe a significant increase in the periodontitis groups, compared to the healthy controls (Alshail et al., 2016; Di Lenardo et al., 2019). During the experiment, a significant increase in CK levels was observed after 21 days of ligature placement compared to the 42-day group, which can be associated with the initial stage of the periodontal inflammatory response, translated in a significant tissue destruction.

Regarding the expression of GGT, it would be expected to observe a significant increase in the periodontitis groups, since it has been implicated as an oxidative marker in the onset and progression of periodontitis (Sreeram et al., 2015; Di Lenardo et al., 2019). However, only a slight increase in the expression of this biomarker was observed in the 42-day group, compared to the other experimental and control groups, which may be related to the increased oxidative stress.

The other biochemical marker that has been recently associated with the progression of clinical periodontitis is the enzyme LDH. A higher concentration of this enzyme is usually associated with cell necrosis and tissue degradation, which are closely related to the pathogenesis of this disease (Ali et al., 2018; Moghadam et al., 2022). Our results showed that a significant increase in LDH levels was detected in the 42- and 56-day periodontitis-induced groups when compared to the corresponding controls, indicating a possible severe stage of periodontitis. Importantly, it has been reported that infertile women usually have higher serum levels of LDH, compared to healthy individuals, which may be related to oxidative stress (Veena et al., 2008).

When interpreting the results of this study, an important consideration should be noticed. Indeed, our experiment has some strengths, including the representativeness and global serum coverage analysis, as well as the strict methodology followed for the induction of periodontitis. Nevertheless, there are some limitations to be mentioned. First, the rat periodontal apparatus and host susceptibility are not totally identical to humans, and the rat model used in our experiment do not develop periodontitis spontaneously. It was necessary to place and maintain silk ligatures in the teeth to facilitate bacterial adherence and colonization, mimicking the disease in humans. Nevertheless, the multifactorial nature of human periodontitis is impossible to achieve in animal models (Kinane et al., 2017), since the oral dysbiosis and bacterial accumulation is only one of the risk factors in the onset and progression of periodontitis (Tonetti et al., 2018). Therefore, these aspects should always be considered when interpreting results. Secondly, our results were based on a modest number of animals, highlighting the need to use larger samples, in future studies, to confirm the results obtained throughout this experiment. The reduced sample size is associated with the number of experimental and control groups considered and the 3 R’s principle. Our results indicated that after 42 days of ligature exposure, rats appeared to have chronic periodontitis. However, given the small sample size, further investigations are needed to validate and increase the statistical power analysis. Third, female rats have a very short estrous cycle, and the phase present in each rat was not considered, which could lead to bias in the expression of sex-related hormones, such as estradiol and progesterone (Lovick and Zangrossi, 2021). Fourth, it is also important to consider the evaluation of other inflammatory mediators, such as tumor necrosis factor-α, and interleukin-6, -1β, and −10, which are closely related to the onset and progression of periodontitis (Darveau, 2010; Ebersole et al., 2013). Finally, the ligature induction periodontitis method used in our experiment caused a bone loss in only two maxillary molars, with a localized effect instead of a generalized periodontitis that may have a more severe systemic inflammatory effect.

The results of the present study can play a pivotal role for the research and medical communities, as it will be the first time, to our knowledge, that the potential biological mechanisms involved in the relationship between periodontitis and female infertility are analyzed.

As future perspectives, the development of prospective and/or retrospective studies in a clinical setting should be considered, increasing the current evidence about potential risk factors associated with this link. In a non-clinical position, it would be interesting to use this rat model of periodontitis to further study the fertility rate and potential pregnancy complications, as well as the biological mechanisms involved, such as cell signaling and gene regulation.

## 5 Conclusion

This rat model of ligature-induced periodontitis showed important morphometric and serological differences up to 56 days of exposure to ligature. These results will allow for future comparability standards and progress our knowledge in of the link between periodontitis and female fertility in preclinical research.

## Data Availability

The original contributions presented in the study are included in the article/Supplementary Material, further inquiries can be directed to the corresponding author.
